# Identification of Candidate Heat-Tolerance Genes in Maize by Integrating Linkage and Transcriptomic Analyses

**DOI:** 10.3390/plants15050691

**Published:** 2026-02-25

**Authors:** Mei Han, Xianfeng Yang, Jingfu Ma, Yuanming Wu, Chang Wang, Xingrong Wang, Yunling Peng, Yanjun Zhang

**Affiliations:** 1 College of Agronomy, Gansu Agricultural University, Lanzhou 730070, China; 14719448664@163.com (M.H.); 13919294227@163.com (Y.W.); wxr_0618@163.com (X.W.); 2 State Key Laboratory of Aridland Crop Science, Gansu Agricultural University, Lanzhou 730070, China; 3 Crop Research Institute, Gansu Academy of Agricultural Sciences, Lanzhou 730070, China; 13110501226@163.com (X.Y.); 15193167129@163.com (J.M.); chang288@163.com (C.W.); 4 Key Laboratory of Crop Gene Resources and Germplasm Innovation in Northwest Cold and Arid Regions, Ministry of Agriculture and Rural Affairs, Lanzhou 730070, China

**Keywords:** maize, heat stress, QTL mapping, transcriptomic analysis, candidate genes

## Abstract

With global warming, high-temperature stress has become a primary abiotic factor limiting maize yield and quality. Exposure to heat stress induces sunscald on maize leaves, which severely impairs photosynthesis and ultimately leads to yield reduction. In this study, we used the heat-tolerant inbred line Zheng58 and the heat-sensitive inbred line HSBN, both of which are cultivated maize (*Zea mays* L. subsp. *mays*) inbred lines, as parents to construct F_2_ and F_2:3_ populations consisting of 257 lines. Phenotyping for sunscald at the flowering stage was performed across three field environments. The F_2_ population was genotyped using the Maize 10K SNP array to construct a genetic map containing 1728 single nucleotide polymorphism (SNP) markers. The map spanned 1406.22 cM, with an average marker density of 0.81 cM per marker. Eight quantitative trait loci (QTLs) associated with heat tolerance were identified in the F_2_/F_2:3_ populations, distributed on chromosomes 1, 4, 5, and 8, collectively explaining 3.43% to 35.44% of the phenotypic variation. Among them, the stable QTL *qHT1-2* on chromosome 1 was consistently detected across all three environments, explaining 11.41% to 35.44% of the phenotypic variation. Additionally, a major QTL, *qHT1-3*, was identified on the same chromosome, accounting for 33.70% of the phenotypic variation. Transcriptome analysis of flowering-stage leaves from both parents revealed 9262 differentially expressed genes (DEGs). Of these, 21 DEGs were co-localized within the eight QTL intervals. The genes *Zm00001eb013260*, *Zm00001eb012720*, *Zm00001eb013600*, and *Zm00001eb013100* exhibited highly significant differential expression between the parental lines, these four genes are identified as candidate genes in response to heat stress in maize, and their specific biological functions require further functional validation.

## 1. Introduction

Amidst the intensifying global warming, the frequency and intensity of heatwaves continue to increase, posing a severe threat to China’s food security [1]. As a primary food and feed crop, the stable production of maize is crucial for ensuring food security. High temperatures during the flowering stage of maize can reduce pollen viability and hinder pollination and fertilization, ultimately leading to significant yield losses [2]. Therefore, a thorough understanding of the genetic mechanisms underlying heat tolerance during flowering is of strategic importance for breeding new maize varieties adapted to high-temperature environments and for ensuring global food security. As a thermophilic crop, maize thrives within an optimal temperature range of 25–35 °C. Prolonged exposure to temperatures above 35 °C significantly impairs plant growth and reproductive development [3]. Research indicates that high temperatures are often accompanied by an elevated vapor pressure deficit, which intensifies transpiration and water loss. When combined with soil moisture deficit, this creates a more severe compound “heat-drought” stress [3]. This condition decreases the relative water content and water potential of leaves, leading to wilting, curling, or even desiccation [4]. High temperatures also accelerate chlorophyll degradation, either directly or by inducing oxidative stress, resulting in reduced chlorophyll content, chlorosis, or bleaching [5,6,7,8]. This phenomenon not only diminishes the light-harvesting capacity but also serves as a direct indicator of damage to the photosynthetic apparatus. Photosystem II (PSII) is among the most heat-sensitive components of photosynthesis, and its impairment can be precisely monitored through chlorophyll fluorescence parameters [9,10,11].

Leaf scorch (sunburn) during maize flowering is a key phenotypic indicator for assessing heat tolerance, reflecting physiological and molecular disturbances induced by thermal stress. Physiologically, high temperatures disrupt ROS metabolic homeostasis, leading to oxidative damage of cell membranes and biomacromolecules, while leaf dehydration and impaired photosynthesis exacerbate necrosis [12]. At the molecular level, stress signal transduction, antioxidant defense, and programmed cell death suppression genes collectively regulate scorch occurrence. ABA activates sucrose transporters and synthases (providing energy/signal), whereas hormones such as SA, JA, and BR modulate antioxidant systems and stomatal movement, forming a complex regulatory network for heat stress adaptation [13].

Given the severe impact of high-temperature stress on maize production—particularly during the flowering stage—understanding heat tolerance is essential. This trait is a complex quantitative characteristic controlled by multiple genes and exhibits significant phenotypic variation among cultivars. Due to its genetic complexity, quantitative trait locus (QTL) mapping and transcriptomic analysis have become the most widely employed approaches to elucidate the molecular genetic mechanisms underlying heat tolerance in maize [14].

Leaf scorch at the flowering stage serves as a key phenotypic indicator for assessing maize heat tolerance, and its genetic basis has been partially explored in previous studies. For instance, Ries et al. identified six QTLs associated with nine heat-sensitivity indices in temperate maize at the seedling stage, with each QTL explaining 7% to 9% of the phenotypic variation [15]. Zhang et al. constructed a high-density genetic map via SLAF-seq using a recombinant inbred line (RIL) population derived from the heat-tolerant inbred line Abe2 and heat-sensitive inbred line B73, and further detected four QTLs related to flowering-stage leaf scorch on chromosomes 1, 2 (two QTLs), and 3. These QTLs collectively accounted for approximately 19.73% of the phenotypic variation in the trait, and subsequent fine-mapping of the qLS1 locus on chromosome 1 combined with QTL-seq data pinpointed six candidate genes [16].

Transcriptome analysis has also been extensively applied to dissect the transcriptional response network of maize to heat stress. Through integrated transcriptomics and weighted gene co-expression network analysis (WGCNA) of the heat-tolerant inbred line *CIMBL55*, Long et al. identified core regulatory factors including key transcription factors, heat shock proteins, Ca^2+^ signaling-related genes, and several enzymatic hub genes [17]. Among these, the hub gene *ZmHsftf13* was functionally validated via heterologous expression in Arabidopsis and insertion mutagenesis in maize, confirming its positive regulatory role in mediating maize heat tolerance. While both linkage analysis and transcriptomics are effective strategies for identifying heat tolerance-associated candidate genes, each approach has inherent limitations: linkage analysis is confined to the allelic variation present in two specific parental lines, and the precision of QTL mapping is constrained by marker density, often preventing precise gene localization; transcriptomic analysis, in contrast, captures genome-wide expression changes under stress, directly reflecting the dynamic responses of functional genes and providing expression-level validation for candidates identified by QTL mapping. This integrated application of the two approaches can reduce the analytical bias inherent in single-method studies. For example, after mapping the major kernel length QTL *qKL-2* in maize, Wang et al. identified 11 differentially expressed protein-coding genes within a 1.95 Mb interval by comparing parental expression profiles, thereby refining the candidate gene list [18]. Similarly, Chen et al. performed meta-analyses of 839 blast resistance and 308 heat tolerance QTLs in rice, and combined the meta-QTL data with differential expression analysis to identify co-localized candidate genes (*OsChib3H-c*, *OsJAMyb*, *Pi-k*) that mediate plant responses to both biotic and abiotic stresses [19].

Despite these advances, few studies have integrated both linkage mapping and transcriptomic strategies to genetically dissect quantitative traits associated with heat tolerance during maize flowering. In this study, we characterized sunscald (leaf scorch) in maize at the flowering stage across multiple environments. Using a linkage map constructed from 1728 SNP markers, we performed QTL mapping for high-temperature stress responses during flowering. This was integrated By analyzing the transcriptomic data of maize leaves at the flowering stage, we adopted a synergistic screening strategy to identify heat-tolerant candidate genes. As the primary photosynthetic and stress-sensing organs of maize, leaves play a pivotal role in mediating the whole-plant response to high-temperature stress and are critical for sustaining the normal development of reproductive organs during the flowering period. Based on this physiological relevance, the present study used leaf transcriptomic data to identify core heat-tolerant genes associated with the maintenance of the flowering process under high-temperature stress. Our work aims to identify robust candidate genes underlying heat tolerance in maize at the flowering stage by integrating leaf molecular responses with flowering-stage phenotypic performance, thereby providing genetic resources and material support for elucidating the molecular mechanisms of heat tolerance and advancing molecular breeding for heat tolerance in maize.

## 2. Results

### 2.1. Phenotypic Statistical Analysis

Analysis of leaf sunscald traits in parental lines and F_2_/F_2:3_ populations across two experimental locations (Zhangye and Dunhuang) showed that HSI-DH (F_3_ population in Dunhuang, 2025) exhibited the most severe sunscald index, with a value of 19.04. This was significantly higher than the values of HSI-ZY (F_3_ population in Zhangye, 2025, 5.43) and HSI-ZYF_2_ (F_2_ population in Zhangye, 2024, 1.48). Significant differences in sunscald severity were detected between the two environments after high-temperature stress treatment (Table 1). Estimates of broad-sense heritability (h^2^) indicated that the HSI-ZYF_2_ population had the highest heritability (36.75%), whereas HSI-ZY and HSI-DH showed relatively lower heritability (approximately 18%). These results suggest that the expression of this heat tolerance-related trait in maize is strongly influenced by environmental factors.

Leaf sunscald is a typical quantitative trait jointly regulated by genetic and environmental factors; while the relatively low heritability of this trait warrants consideration of its phenotypic stability for genetic analysis, it remains a valid and biologically relevant indicator for dissecting the genetic basis of maize heat tolerance. This validity is supported by two key experimental observations: significant phenotypic divergence in sunscald severity between the parental lines, and a detectable genetic component (18–37% *h*^2^) across all populations. These two features collectively provide a sufficient genetic foundation for the identification of stable quantitative trait loci (QTLs) underlying maize heat tolerance to high-temperature stress.

**Figure 1:**
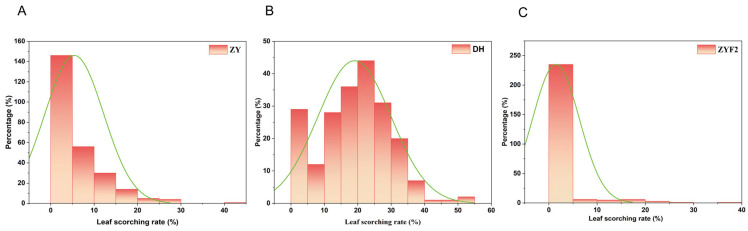
Leaf sunscald rate after high-temperature treatment in different populations. (**A**) Zhangye (ZY) parental line; (**B**) Dunhuang (DH) parental line; (**C**) Zhangye F_2_ population (ZYF_2_). green line Follows a normal distribution.

The normality distribution test results (Figure 1) and corresponding phenotypic statistics (Table 1) show that the leaf scorching rate (reflected by HSI, heat stress index) in the F_2_ population (HSI-ZYF_2_) exhibits continuous variation with a wide range (0–35.94%) and a high coefficient of variation (CV = 3.29%) (Table 1). Meanwhile, the distribution of HSI-ZYF_2_ in Figure 1 shows a distinct right skew (Skew = 3.99) and sharp peak (Kurt = 17.44), which differs from the relatively concentrated, low-skew distributions of the parents (e.g., HSI-ZY: Skew = 1.61; HSI-DH: Skew = 0.07, Table 1). Such continuous phenotypic segregation (instead of discrete bimodal distribution) and the high skewness/kurtosis of the F_2_ population are typical hallmarks of complex quantitative traits—these characteristics arise from the cumulative effects of multiple genetic loci. Thus, we infer that maize heat tolerance follows a quantitative inheritance model involving a major gene and multiple minor genes. The F_2_ population also shows a moderate heritability (*h*^2^ = 36.75%, Table 1), which further confirms the genetic stability of this trait and provides reliable phenotypic and genetic basis for subsequent QTL mapping and candidate gene screening of maize heat tolerance.

### 2.2. Construction of Genetic Maps

**Figure 2:**
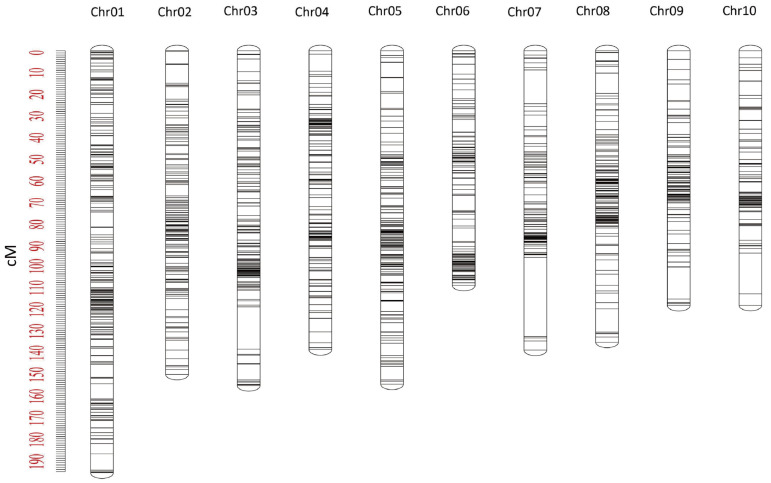
SNP High-Density Genetic Linkage Map. Red numbers represents genetic distance.

In this study, genomic DNA from 257 F_2_ individuals was genotyped using the Maize 10K SNP array. A total of 10,005 SNP markers were obtained, and after quality control (MAF ≥ 0.05, missing rate < 0.10), 1728 high-quality SNPs were retained for genetic linkage map construction (Figure 2; Table 2). Among the ten chromosomes, chromosome 1 contained the largest number of markers (264), whereas chromosome 10 had the fewest (113). Using MapMarker 3.0 software, the 1728 SNPs were assigned to ten linkage groups, with individual group lengths ranging from 108.27 cM to 194.48 cM. Marker density varied from 0.96 to 1.36 cM per marker, and the total map length was 1406.22 cM.

### 2.3. QTL Mapping Results

**Figure 3:**
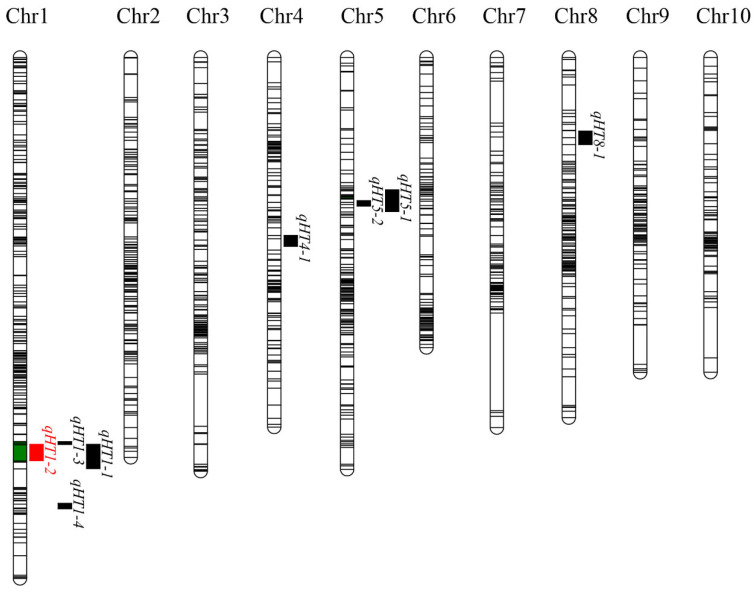
QTL map for sunscald traits in the maize F_2_ population. The red color represents stable QTL detected in three environments; the black color represents QTL detected in one to three environments. Green color represents both stable and other QTLs were detected in this interval.

Based on phenotyping data for leaf scorch traits at flowering across three environments in the F_2_/F_2:3_ population, quantitative trait locus (QTL) mapping was performed. Eight QTLs were identified: seven using the ICIM method and one using the IM method (Figure 3). These included *qHT1-1*, *qHT1-2*, *qHT1-3*, *qHT1-4*, *qHT4-1*, *qHT5-1*, *qHT5-2*, and *qHT8-1* (Table 3). Their logarithm of odds (LOD) scores ranged from 2.53 to 29.79, and they explained 3.43–35.44% of the phenotypic variance (PVE: phenotypic variation explained). Among them, the stable QTL *qHT1-2* was detected in multiple environments and explained 35.44% of the phenotypic variance, whereas the major-effect QTL *qHT1-3* explained 33.70% of the variance in a single environment (ZYF_2_). Six of these QTLs carried favorable alleles from the heat-tolerant parent Zheng58 (Table S1).

### 2.4. Transcriptome Analysis

#### 2.4.1. Transcriptome Sequencing Quality Assessment

**Figure 4:**
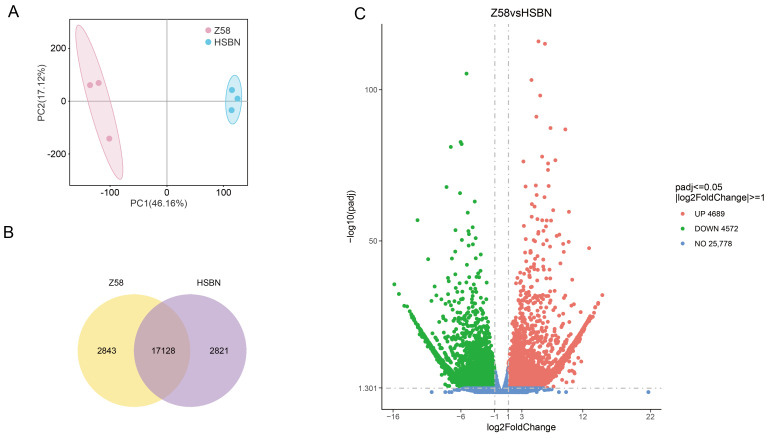
Transcriptome analysis of maize under high-temperature stress during flowering. (**A**) Read density distribution plots for Zheng58 and HSBN; (**B**) Venn diagram of differentially expressed genes (DEGs), showing numbers of up- and down-regulated genes; (**C**) Volcano plot of gene expression changes. Each point represents a gene. The x-axis indicates the log_2_ fold change (log_2_FC) between samples, and the y-axis shows the −log_10_ of the adjusted *p*-value. Red points denote up-regulated DEGs, green points indicate down-regulated DEGs, and blue points represent genes with no significant differential expression.

Summary statistics of the transcriptome sequencing data for the parental materials are provided in (Table S2). Across all samples, the percentages of Q20 and Q30 bases exceeded 99.23% and 96.62%, respectively, and the GC content ranged from 54.8% to 55.65%. Principal component analysis (PCA) showed clear separation among biological replicates from different parents, whereas replicates from the same parent clustered closely together (Figure 4A), indicating pronounced transcriptional differences between genotypes and high reproducibility within each group. These results support the overall reliability of the sequencing data. Following alignment of the HISAT2-processed clean reads to the maize reference genome (Zm B73_REFGEN_ v5a), consistent genome coverage was observed across all samples, further confirming uniform data quality. In summary, both the quality and quantity of the transcriptomic sequencing data meet the standards required for subsequent analyses.

#### 2.4.2. Analysis of Differentially Expressed Genes in Leaves Under High-Temperature Stress

**Figure 5:**
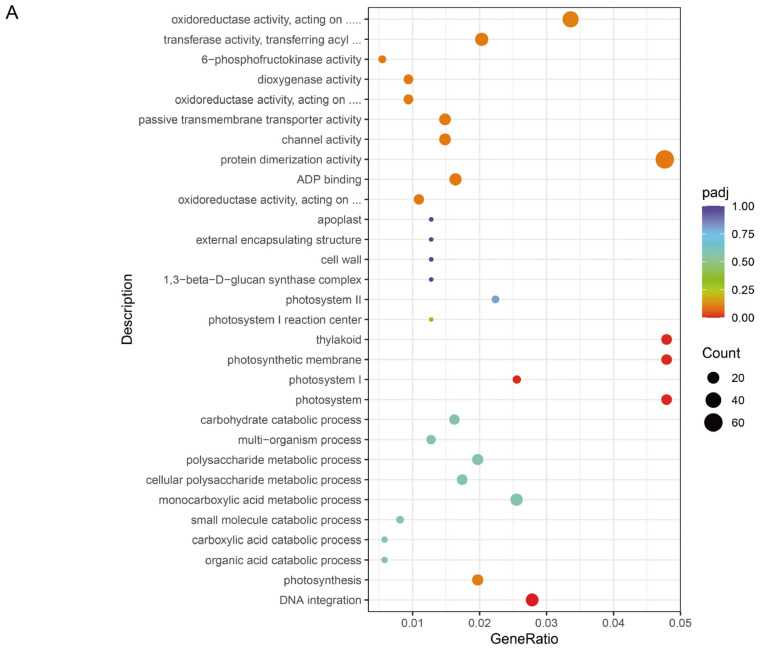

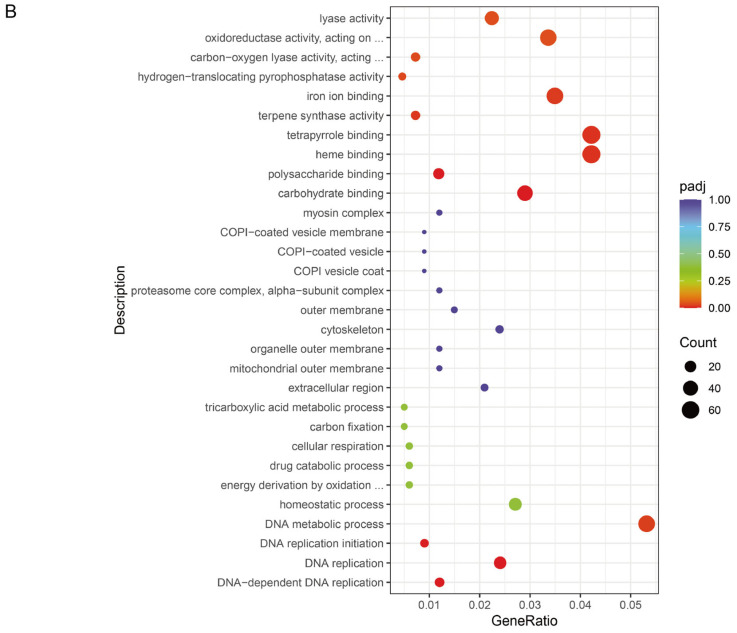
Gene Ontology (GO) enrichment analysis of differentially expressed genes. (**A**) Significantly enriched GO terms among up-regulated genes; (**B**) Significantly enriched GO terms among down-regulated genes.

**Figure 6:**
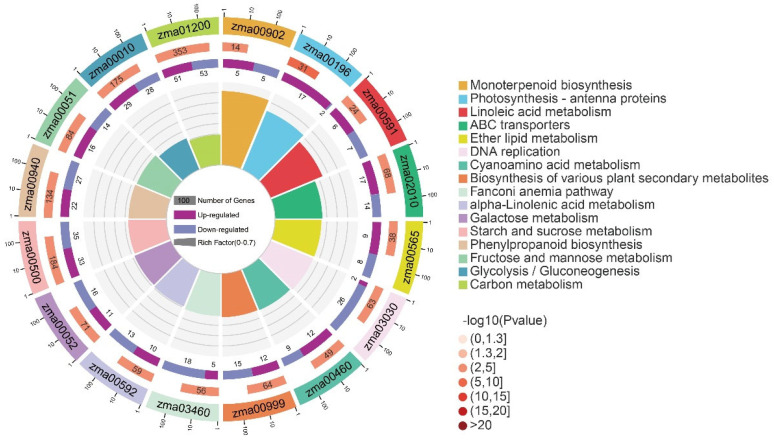
KEGG pathway enrichment analysis of differentially expressed genes. The four concentric circles (from inside to outside) represent the following information: the first circle shows enriched KEGG pathways and the number of genes mapped to each; the second circle displays the total number of genes in the genomic background for each pathway and the enrichment significance (*p*-value); the third circle indicates the number of up-regulated (dark purple) and down-regulated (light purple) genes; the fourth circle represents the gene ratio, i.e., the proportion of differentially expressed genes within each pathway.

A total of 17,128 genes were found to be co-expressed between the heat-tolerant genotype Zheng58 and the heat-sensitive genotype HSBN (Figure 4B). Among these, 9261 genes were identified as differentially expressed genes (DEGs), consisting of 4689 up-regulated and 4572 down-regulated genes (Figure 4C). To explore the functions of these DEGs, Gene Ontology (GO) enrichment analysis was conducted separately on the up-regulated and down-regulated gene sets. Enriched terms spanned three main categories: biological process (BP), cellular component (CC), and molecular function (MF). The up-regulated genes were primarily associated with cellular components related to photosynthesis, including photosystem I, photosynthetic membrane, and thylakoid, and were enriched in the molecular function of DNA integration (Figure 5A). The down-regulated genes were mainly enriched in BP terms such as DNA metabolism and DNA replication, and in MF terms including carbohydrate binding, polysaccharide binding, heme binding, iron ion binding, and oxidoreductase activity (Figure 5B). To further clarify the metabolic pathways involved, KEGG pathway enrichment analysis was performed. The results indicated that the DEGs were significantly enriched in pathways related to Photosynthesis, Photosynthesis-antenna proteins, ABC transporters, DNA replication, Homologous recombination, Amino sugar and nucleotide sugar metabolism, and Flavonoid biosynthesis (Figure 6).

### 2.5. Integration of QTL Mapping and Transcriptome Analysis for Candidate Gene Identification

**Figure 7:**
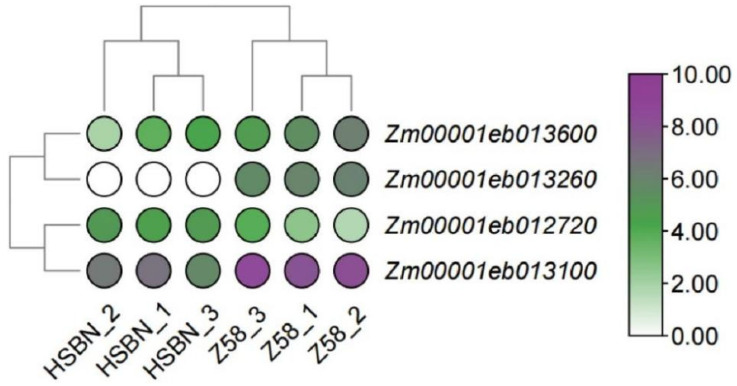
Expression levels of candidate genes for maize heat tolerance.

To further identify key candidate genes involved in the high temperature stress response, the genomic intervals identified by QTL mapping were cross-referenced with the 9261 differentially expressed genes (DEGs) obtained from transcriptome sequencing of the parental lines used in this study. This integrated analysis revealed 21 DEGs located within the QTL regions. Among these, four genes—*Zm00001eb013100*, *Zm00001eb013260*, *Zm00001eb012720*, and *Zm00001eb013600*—showed particularly pronounced expression differences (Figure 7; Table S3). These genes were identified as candidate genes, which require further verification.

### 2.6. Validation of Candidate Genes by qRT-PCR

**Figure 8:**
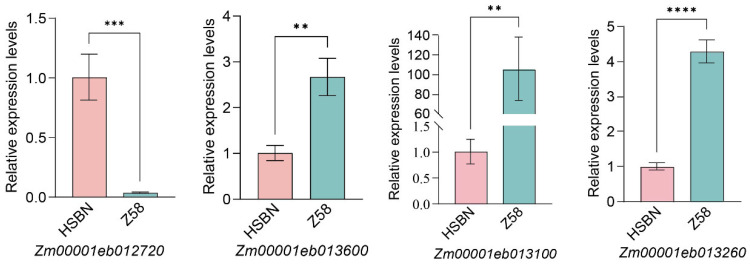
Relative expression levels of candidate genes in heat-tolerant vs. heat-sensitive materials. ns, not significant; ** *p* < 0.01; *** *p* < 0.001; **** *p* < 0.0001.

To validate the expression patterns of the four candidate genes under high-temperature stress, their transcript levels were analyzed by qRT-PCR in the parental lines (Figure 8). After high-temperature treatment, the expression of *Zm00001eb012720* was higher in the heat-sensitive parent HSBN than in the heat-tolerant parent Zheng58. In contrast, the other three genes showed higher expression in the heat-tolerant parent. These results suggest that the four genes are likely involved in the regulation of heat tolerance in maize during flowering.

## 3. Discussion

### 3.1. Environmental Regulation and Genetic Modulation of Heat Tolerance Phenotypes in Maize

Under global warming, frequent extreme high-temperature events in summer have become a major abiotic stress limiting stable maize production and threatening food security in China [20]. Maize is particularly sensitive to heat stress at the flowering stage, which impairs pollen development, fertilization, and grain formation, and causes leaf sunscald that further reduces photosynthetic efficiency [21,22,23].

Heat stress accelerates leaf senescence characterized by reduced green leaf area, leaf desiccation, and chlorophyll degradation [24]. Correspondingly, obvious leaf yellowing, wilting, and sunscald symptoms were observed in our materials under high temperature (Figure 1), which directly reflect heat-induced physiological injury. Sunscald further reduces photosynthetic capacity, damages membrane integrity, and exacerbates leaf senescence.

Genetic background strongly influences heat tolerance in maize. Previous studies indicated that heat shock protein (HSP) expression in hybrids and segregating populations is controlled by major genetic loci with dominant effects [25]. Notably, heat tolerance in plants is regulated by complex signaling networks, among which hormone-mediated pathways play crucial regulatory roles [13]. Plant hormones such as abscisic acid (ABA), jasmonic acid (JA), salicylic acid (SA), and brassinosteroids (BRs) coordinate with each other to mediate the expression of heat-responsive genes, thereby regulating plant heat tolerance. However, the role of plant hormones in the heat stress response of maize was not sufficiently discussed in the previous research, which limits the in-depth interpretation of the genetic and molecular mechanisms underlying maize heat tolerance.

In this study, phenotypic analysis revealed substantial variation in sunscald resistance among populations and environments, indicating a combined control by genetic and environmental factors. The population grown at the Dunhuang site showed much more severe sunscald symptoms than the ZY and ZYF_2_ populations (Table 1), supporting the strong environmental influence on the phenotypic expression of heat tolerance-related traits in maize. This environmental dependence may be closely related to the interaction between hormone signaling pathways and environmental factors. drought-induced ABA accumulation can enhance maize heat tolerance, while high temperature may affect the synthesis and transport of hormones, thereby regulating the occurrence of leaf sunscald, which deserves further exploration in subsequent studies.

### 3.2. QTL Analysis of Heat Tolerance

Heat tolerance in maize is a complex quantitative trait controlled by multiple genetic loci. In this study, a genetic linkage map with a total length of 1406.22 cM was constructed using 1728 single nucleotide polymorphism (SNP) markers. In the F_2_/F_2:3_ population, eight quantitative trait loci (QTLs) associated with heat tolerance were identified on chromosomes 1, 4, 5, and 8, collectively explaining 3.43% to 35.44% of the phenotypic variation. A stable QTL, *qHT1-2*, was mapped to chromosome 1 (physical interval: 41,182,244–46,926,670 Mb), explaining 11.41% to 35.44% of the phenotypic variation across environments. Among the identified QTLs in this study (*qHT1-1*, *qHT1-2*, *qHT1-3*, *qHT1-4*, *qHT4-1*, *qHT5-1*, *qHT5-2*, and *qHT8-1*), several have not been reported previously and may represent novel genetic loci for heat tolerance, providing potential targets for marker-assisted selection. Sunscald, a key indicator of high-temperature stress in maize, is strongly influenced by environmental conditions, consistent with its low to moderate heritability observed in this study. This trait has been studied extensively. For example, Zhang et al. identified four QTLs (*qLS1*, *qLS2.1*, *qLS2.2*, *qLS3*) for leaf scorch on chromosomes 1, 2, and 3, explaining 19.73–31.23% of phenotypic variation, and ultimately pinpointed the candidate gene *Zm00001d033328* via QTL-seq [16]. In a separate study on grapevines under heat stress, Heinekamp et al. identified two major QTLs on chromosomes 10 and 11 explaining 14.1% and 26.1% of phenotypic variation, respectively, highlighting the additive effect of favorable alleles [26]. These studies underscore the value of QTL mapping in dissecting the genetic and environmental architecture of complex heat-responsive traits. the QTLs identified in this study, especially the stable *qHT1-2*, are likely associated with hormone-mediated heat tolerance pathways. As systematically summarized by Li et al. [13]. hormone-mediated signaling is critical for plant heat tolerance, and many heat-responsive QTLs regulate hormone synthesis, transport, or signal transduction—consistent with findings in rice and wheat where QTLs linked to ABA content or signaling correlate with heat tolerance. Thus, our identified QTL intervals may harbor candidate genes involved in hormone-mediated heat stress responses.

### 3.3. Transcriptome Analysis of Maize Leaves Under High-Temperature Stress

This study conducted RNA-seq analysis on leaves of the heat-tolerant selfing line Zheng58 and the heat-sensitive selfing line HSBN collected under natural high-temperature stress. By comparing the transcriptomes of the two genotypes under identical stress conditions, we identified differentially expressed genes and pathways associated with heat-tolerance phenotypes.

High-temperature stress induces extensive transcriptional reprogramming in maize cells, leading to the differential expression of numerous genes involved in heat response, reactive oxygen species scavenging, and protein homeostasis [27]. Systematic studies of these expression changes have progressively uncovered the underlying molecular regulatory networks and key mechanisms [28]. Under heat stress, significant differences in gene expression have been observed between heat-tolerant and heat-sensitive varieties. For instance, Liu et al. reported that in sweet corn, 516 co-upregulated genes were enriched in chloroplast-related processes such as photosynthesis and thylakoid function, highlighting chloroplast proteins as central to heat tolerance. Conversely, 1261 down-regulated genes were associated with basic biological processes including gene expression and translation; their moderate suppression may help conserve energy and enhance stress resilience. Heat-tolerant sweet corn may further adapt by down-regulating genes involved in brassinosteroid synthesis, thereby suppressing growth under stress [29]. Similarly, studies by Wenhe Pan et al. indicate that heat stress strongly inhibits photosynthetic pathways, while heat-tolerant genotypes maintain better photosynthetic function, showing smaller declines in photosystem II efficiency (Fv/Fm) and more moderate changes in photosynthesis-related gene expression [30,31]. In this study, transcriptome analysis revealed that the heat-tolerant line Zheng58 exhibited enrichment in molecular functions related to DNA integration, photosynthetic systems, and ABC transporters. The enrichment in pathways associated with DNA integration could hypothetically imply a potential link to transposon-mediated genome dynamics and adaptive genome plasticity, although this remains speculative given the limitations of transcriptome-based inference.

Upregulation of photosynthesis and ABC transporter pathways suggests that Zheng58 may alleviate heat-induced damage by preserving photosystem integrity, sustaining efficient energy capture and conversion, and maintaining ion and metabolite transport homeostasis.

In contrast, the heat-sensitive line HSBN showed significant down-regulation of biological processes including DNA replication, repair, and related metabolic pathways. Notably, pathways such as DNA replication, homologous recombination, and mismatch repair were strongly activated, indicating severe DNA damage and replication stress. Down-regulation of amino sugar and nucleotide metabolism may relate to cell-wall remodeling under stress, while suppression of flavonoid biosynthesis and iron/heme oxidoreductase activity could reflect a compromised chemical defense system against oxidative damage.

### 3.4. Functional Validation of Candidate Genes

To further elucidate key candidate genes for high-temperature stress response, we cross-referenced the genomic regions identified by QTL mapping with 9261 differentially expressed genes obtained from transcriptome sequencing, jointly screening out 21 candidate genes. Among these, four genes exhibited extremely significant differences. After years of multi-site validation, four candidate genes identified in this study have only been confirmed for their stable heritability in parental materials and significant differential expression under high-temperature stress, while their specific biological functions and regulatory mechanisms in maize heat tolerance remain to be further verified through gene cloning, overexpression, gene editing and other functional experiments.

The integration of QTL mapping with transcriptome analysis has become a powerful strategy for identifying candidate genes underlying complex traits. Lu et al. identified a major heat-tolerance QTL (*qHT1.13*) on chromosome 1, explaining 40.1–59.6% of phenotypic variation. Within its candidate interval, the gene *Csa1G004990* showed higher expression in the heat-tolerant parent, highlighting it as a key candidate [32]. Similarly, Wang et al. combined QTL mapping and QTL-seq to locate major heat-tolerance loci on chromosomes 1 and 2. Subsequent RNA-seq and qRT-PCR validation identified *SlCathB2*, *SlGST*, *SlUBC5*, and *SlARG1* as important candidate genes [33]. In this study, by combining linkage analysis with transcriptomic profiling, four candidate genes for heat tolerance were identified: *Zm00001eb013260*, *Zm00001eb012720*, *Zm00001eb013600*, and *Zm00001eb013100*. *Zm00001eb013100* (43.53–43.53 Mb) encodes mitogen-activated protein kinase 3 (MAPK3/MPK3). Studies in *Arabidopsis* indicate that MPK3 activity correlates with heat tolerance, though its functional role appears species-specific. In *Arabidopsis*, *mpk3* mutants paradoxically exhibit enhanced heat tolerance [34,35]. whereas in other species such as *Vinca* and melon, MPK3 up-regulation is associated with improved heat resistance [36,37]. MPK3 also interacts with ABA signaling to mediate drought-induced heat cross-adaptation [38]. In soybean, GmMPK3 is negatively regulated by the E3 ligase GmSAUL1, and its activation triggers immune responses [39]. In maize, however, the role of MPK3 under heat stress remains unreported. *Zm00001eb012720* (42.02–42.03 Mb) encodes a kinesin-like protein, which functions in intracellular transport and cell division. In *Arabidopsis*, the kinesin-like protein AtKP1 localizes to mitochondria and may regulate mitochondrial dynamics [40]. Another microprotein, AUCSIA-1, interacts with a CENP-E-related motor protein to influence auxin transport and root growth, suggesting kinesin-like proteins may modulate cytoskeletal organization during stress adaptation [41]. While similar mechanisms have been proposed in maize, the specific function of this gene under heat stress awaits further investigation.

*Zm00001eb013260* and *Zm00001eb013600* have not been previously reported in the context of heat stress. In this study, both genes were significantly upregulated under high temperature conditions, and their expression levels correlated positively with heat tolerance across maize materials. These findings suggest that *Zm00001eb013260* and *Zm00001eb013600* may represent novel members of the maize heat stress response network, with potential roles in thermotolerance regulation. Four genes were identified as stably inherited candidate genes, which require further verification.

## 4. Materials and Methods

### 4.1. Test Materials

This study used the heat-tolerant inbred line Zheng58 (as female parent) and the heat-sensitive inbred line HSBN (as male parent), both of which are cultivated maize (*Zea mays* L. subsp. *mays*) inbred lines, to construct an F_2_/F_2:3_ population consisting of 257 lines. All experimental materials were developed and supplied by the Crop Research Institute of the Gansu Academy of Agricultural Sciences.

### 4.2. Field Trials and Trait Surveys

**Figure 9:**
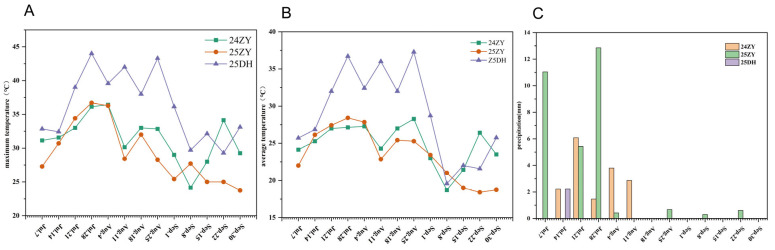
Dynamic changes in daily maximum temperature and precipitation of the maize F_2:3_ family under different environmental conditions. (**A**) Maximum temperature; (**B**) Average temperature; (**C**) Precipitation. HSI-ZY: F_3_ families planted in Zhangye in 2025; HSI-DH: F_3_ families planted in Dunhuang in 2025; HSI-ZYF_2_: F_2_ population planted in Zhangye in 2024.

Phenotypic evaluation of sunburn traits at flowering was conducted in Zhangye (ZY; 38.8501° N, 100.3544° E) and Dunhuang (DH; 40.1630° N, 94.6448° E) in Gansu Province during the 2024–2025 growing seasons. The experimental sites are characterized by a typical temperate semi-arid monsoon climate. The flowering stage of maize occurs from July to September, which is also the period with the highest temperatures. Monthly precipitation in Zhangye was 0–15 mm in 2024, 0–35 mm in 2025, and 0–8 mm in Dunhuang in 2025. The daily maximum temperature ranged from 22 to 40 °C in Zhangye in 2024, 20 to 40 °C in Zhangye in 2025, and 25 to 45 °C in Dunhuang in 2025. In 2024, there were 23 days with the maximum temperature exceeding 35 °C in Zhangye. In 2025, there were 19 such days in Zhangye and 45 days in Dunhuang. The daily average temperature ranged from 15 to 32 °C in Zhangye in 2024, 15 to 31 °C in Zhangye in 2025, and 16 to 39 °C in Dunhuang in 2025 (Figure 9). A randomized complete block design was adopted with a row length of 5 m, row spacing of 0.5 m, and plant spacing of 0.25 m. The F_2_ population was planted without replication, while the F_2:3_ population was evaluated with three replications. To avoid drought stress, irrigation was applied at a rate of 80 m^3^ per mu due to low precipitation (Table S4).

**Figure 10:**
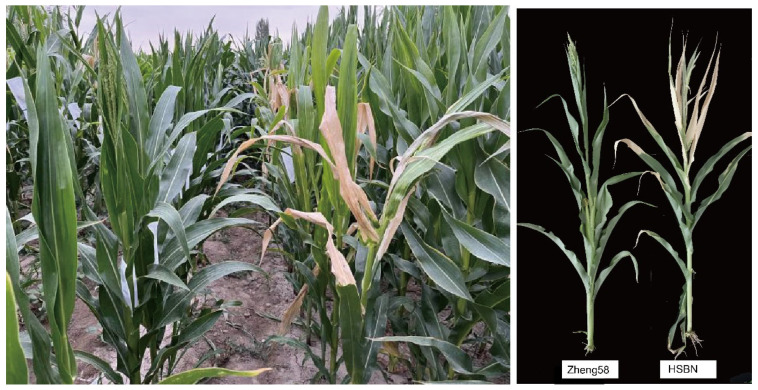
Field phenotype of the parental lines under high temperature stress at the flowering stage. Zheng58 (heat-tolerant) and HSBN (heat-sensitive) plants at the flowering stage, showing leaf senescence and scorching under natural high temperature stress in the field.

Flowering dates were recorded for each line within the parental, F_2_, and F_2:3_ populations (Figure 10), after which heat tolerance was evaluated using a 9-point scale ranging from 1 (mild injury) to 9 (severe injury) [42]. The scoring procedure involved counting the total number of leaves and the number of scorched leaves per plant, followed by calculation of the heat-induced damage rate and the heat damage index to determine the heat-tolerance level. The criteria for rating leaf scorch severity and the formula for calculating the heat-induced damage rate per line are as follows:

Heat-induced damage rate = (Total number of damaged leaves ÷ Total number of leaves) × 100%

The sunscald resistance of maize in this study was investigated, scored and graded strictly according to the grading standards shown in Table 4.

### 4.3. Genotyping and Genetic Mapping

Genomic DNA was extracted from the F_2_/F_2:3_ populations and parental lines using a modified CTAB method [43]. Genotyping was performed with the Maize 10K SNP array (Novogene, Beijing, China). The resulting SNP markers were filtered by first removing loci that were non-polymorphic between the two parents. Subsequently, markers were retained only if they met the following quality thresholds: a minimum allele frequency (MAF) ≥ 0.05, a call rate ≥70%, and a heterozygosity rate ≤20%. This filtering yielded a set of high-quality polymorphic SNPs for map construction. Finally, a genetic linkage map was generated using IciMapping v4.2 [44].

### 4.4. QTL Mapping for Heat Tolerance-Related Traits

QTL mapping was conducted using the Inclusive Composite Interval Mapping (ICIM) and Interval Mapping (IM) methods implemented in IciMapping v4.2 software. The analysis used a scanning step of 1 cM and a probability in stepwise regression (PIN) of 0.001. The LOD significance threshold was set to 2.0, as determined by 1000 permutation tests [45,46]. Detected QTLs were named according to the trait abbreviation, the chromosome number, and the sequential order of the QTL on that chromosome. For instance, *qHT1-1* represents the first QTL identified on chromosome 1. In this study, a QTL was considered “stable” if it was consistently detected across multiple environments or genetic backgrounds with minimal genotype-by-environment interaction, emphasizing its reproducibility. A QTL was classified as “major” based primarily on its high phenotypic variation explained (PVE > 10%) and significant additive effect.

### 4.5. Transcriptome Sequencing and Candidate Gene Screening

To identify differentially expressed genes associated with heat tolerance, this study utilized the heat-tolerant inbred line Zheng 58 and the heat-sensitive inbred line HSBN under natural high-temperature stress as materials. Leaf samples were collected during the flowering period at 14:00 in the afternoon, when the maximum daily temperature exceeded 35 °C and the plants exhibited significant scorch symptoms (as determined by Table 4: Corn High-Temperature Scorch Grading Criteria). From each sampled plant, mid-stage leaf tissues of the 12th to 14th fully expanded leaves were uniformly collected, with three independent biological replicates per genotype. The samples were immediately frozen in liquid nitrogen and stored at−80 °C until RNA extraction.

Leaf samples from both parental lines at the flowering stage were collected under field high-temperature stress (three biological replicates) and subjected to transcriptome sequencing (Beijing Novogene Co., Ltd.). Differential expression analysis between the parental lines was performed using DESeq2 (v1.16.1). Genes were considered differentially expressed if they met the following thresholds: |log_2_(fold change)| ≥ 1 and false discovery rate (FDR) ≤ 0.05. Functional annotation of the identified differentially expressed genes (DEGs) was conducted using the MaizeGDB (https://www.maizegdb.org/) and NCBI (https://www.ncbi.nlm.nih.gov/) databases. GO enrichment analysis and KEGG pathway annotation for DEGs located within stable QTL regions were performed using the online platform GDI Bioinformatics Cloud Tools (https://www.omicshare.com/tools (accessed on 7 January 2025)).

### 4.6. Real-Time Fluorescent Quantitative PCR (qRT-PCR) Analysis

qRT-PCR was performed to validate selected differentially expressed genes. The qRT-PCR reactions were carried out on a ROCHE LightCycler 96 real-time PCR system (Roche Diagnostics, Mannheim, Germany). Total RNA was isolated from the 12th to 14th fully expanded leaves of plants at the flowering stage when the ambient temperature reached 35 °C and reverse-transcribed into cDNA using a commercial reverse transcription kit (TakaRa RT Kit, Tiangen Agricultural Technology Co., Ltd. Dalian, China). ZmActin was used as the internal reference gene. The qPCR reaction mixture was prepared as follows: 2 μL cDNA template, 0.8 μL each of forward and reverse primer, 10 μL TB Green Premix, and 6.4 μL ddH_2_O. The relative expression levels of the candidate genes were calculated using the 2^(-ΔΔCt) method [47] (Table S5).

## 5. Conclusions

This study performed genetic analysis of sunscald-related traits under high-temperature stress using F_2_/F_2:3_ populations derived from contrasting parental lines. By integrating QTL mapping with transcriptome profiling, we systematically identified major QTLs and differentially expressed genes associated with heat tolerance, leading to the identification of candidate genes that await further functional validation. Overall, this work provides a theoretical foundation and valuable genetic resources for molecular breeding aimed at developing heat-tolerant, high-yielding maize.

First, a genetic linkage map spanning 1406.22 cM was constructed using 1728 high-quality SNP markers. Phenotypic data on leaf scorch at flowering were collected from the F_2_/F_2:3_ population across multiple environments. QTL analysis using both ICIM and IM methods detected eight loci, among which qHT1-2 was identified as a stable QTL detected across environments.

Second, transcriptome sequencing of parental lines under high-temperature stress enabled the screening of differentially expressed genes (DEGs). By cross-referencing DEGs with QTL intervals, four candidate genes—*Zm00001eb013260*, *Zm00001eb012720*, *Zm00001eb013600*, and *Zm00001eb013100*—were prioritized for their significant association with sunscald tolerance. These genes, some of which have been implicated in heat stress responses in maize or other species, provide a functional basis for subsequent validation and potential use in molecular breeding.

## Figures and Tables

**Table 1:** Phenotypic statistics and heritability analysis of target traits across locations.

Site	Average	SD	Skew	Kurt	Min	Max	CV (%)	*h*_2_ (%)
HSI-ZY	5.42	6.76	1.61	3.62	0	42.48	1.24	18.86
HSI-DH	19.03	10.98	0.07	0.13	0	54.76	0.57	17.60
HSI-ZYF_2_	1.47	4.86	3.99	17.44	0	35.94	3.29	36.75

HSI-ZY: F_3_ families planted in Zhangye in 2025; HSI-DH: F_3_ families planted in Dunhuang in 2025; HSI-ZYF_2_: F_2_ population planted in Zhangye in 2024. SD: Standard Deviation; CV: Coefficient of Variation; *h*_2_: Generalized Heritability.

**Table 2:** Summary of genotyping markers in the maize population.

Chromosome	Original SNP	Filtered SNP	Physical Length (cM)	Marker Density (cM/Marker)
1	1559	264	194.48	1.36
2	1032	184	149.33	1.23
3	1152	184	154.50	1.19
4	1093	171	137.97	1.24
5	1093	202	153.84	1.31
6	835	143	108.27	1.32
7	844	136	138.16	0.98
8	926	182	134.52	1.35
9	777	150	117.57	1.28
10	694	113	117.54	0.96
Total	10,005	1728	1406.22	1.23

**Table 3:** QTL mapping results for heat tolerance-related traits.

Trait Name	Chromosome	QTL	Position	Left Marker	Right Marker	LOD	PVE	Add
			(cM)				(%)	
HSI-DH	1	*qHT1-1*	151	1_40807815	1_40424945	6.77	13.84	5.5
HSI-ZYF_2_	1	*qHT1-2*	146	1_46926670	1_41182244	23.83	35.44	3.9
HSI-ZYF_2_	1	*qHT1-3*	144	1_47457646	1_47301674	29.79	33.7	4.36
HSI-ZYF_2_	1	*qHT1-4*	167	1_24941890	1_22739811	3.85	3.43	−1.49
HSI-DH	4	*qHT4-1*	67	4_163646242	4_162678166	3.67	6.72	3.76
HSI-DH	5	*qHT5-1*	52	5_193160482	5_191774898	2.53	4.38	2.76
HSI-ZY	5	*qHT5-2*	54	5_189290545	5_188454941	3.09	3.97	1.51
HSI-ZY	8	*qHT8-1*	32	8_166564914	8_165681725	3.87	5.36	2.11

HIS-ZY: Zhangye; HIS-DH: Dunhuang; HIS-ZYF_2_: Zhangye F2 population; LOD: Logarithm of Odds; PVE: Phenotypic Variation Explained; ADD: Additive Effect.

**Table 4:** Grading standards for high-temperature induced sunscald in maize.

Grade	Resistance Level	Description
1	Highly resistant	High-temperature sunscald rate: 0–25%
3	Resistant	High-temperature sunscald rate: 25–50%
5	Moderately resistant	High-temperature sunscald rate: 50–75%
7	Susceptible	High-temperature sunscald rate: 75–100%
9	Highly susceptible	High-temperature sunscald rate: 100%

## Data Availability

The data are contained within the article.

## Supplementary Materials

The following supporting information can be downloaded at: https://ngdc.cncb.ac.cn/gsa/browse/CRA038821, accessed on 22 February 2026. Table S1: QTLs commonly identified by the ICIM and IM methods. Table S2: Statistics of transcriptome sequencing data volume and quality assessment. Table S3: Functional annotation results of candidate genes. Table S4: Meteorological data from 2024 to 2025. Table S5: Specific primer design schemes for candidate genes.
